# Sulfate and Molybdate Incorporation at the Calcite–Water Interface: Insights from Ab Initio Molecular Dynamics

**DOI:** 10.1021/acsearthspacechem.1c00131

**Published:** 2021-07-27

**Authors:** Scott D. Midgley, Devis Di Tommaso, Dominik Fleitmann, Ricardo Grau-Crespo

**Affiliations:** † Department of Chemistry, University of Reading, Whiteknights, Reading RG6 6DX, U.K.; ‡ Department of Chemistry, School of Biological and Chemical Sciences, Queen Mary University of London, Mile End Road, London E1 4NS, U.K.; § Department of Archaeology, University of Reading, Whiteknights, Reading RG6 6AB, U.K.; ∥ Department of Environmental Sciences, University of Basel, Bernoullistrasse 32, Basel 4056, Switzerland

**Keywords:** calcite, sulfate, molybdate, stalagmites, volcanic activity records, *ab initio* molecular dynamics

## Abstract

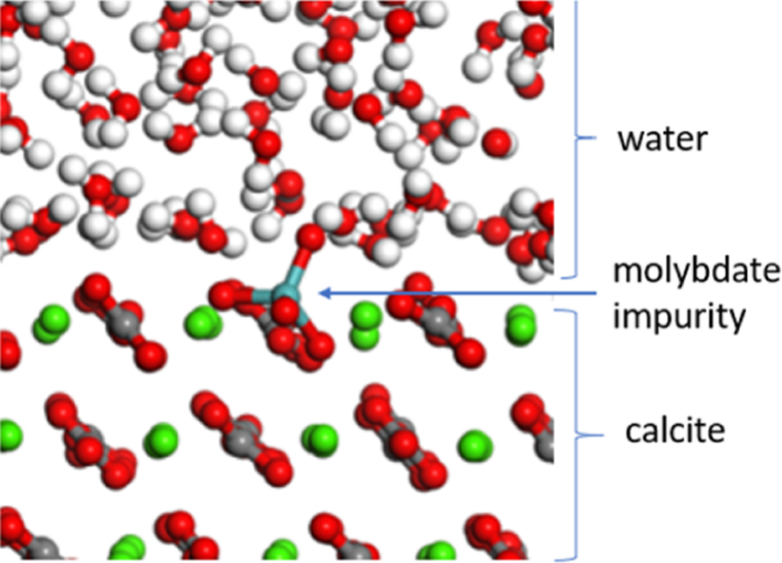

Sulfur and molybdenum trace impurities in speleothems (stalagmites and stalactites) can provide long and continuous records of volcanic activity, which are important for past climatic and environmental reconstructions. However, the chemistry governing the incorporation of the trace element-bearing species into the calcium carbonate phases forming speleothems is not well understood. Our previous work has shown that substitution of tetrahedral oxyanions [*X*O_4_]^2–^ (*X* = S and Mo) replacing [CO_3_]^2–^ in CaCO_3_ bulk phases (except perhaps for vaterite) is thermodynamically unfavorable with respect to the formation of competing phases, due to the larger size and different shape of the [*X*O_4_]^2–^ tetrahedral anions in comparison with the flat [CO_3_]^2–^ anions, which implied that most of the incorporation would happen at the surface rather than at the bulk of the mineral. Here, we present an ab initio molecular dynamics study, exploring the incorporation of these impurities at the mineral–water interface. We show that the oxyanion substitution at the aqueous calcite (10.4) surface is clearly favored over bulk incorporation, due to the lower structural strain on the calcium carbonate solid. Incorporation at surface step sites is even more favorable for both oxyanions, thanks to the additional interface space afforded by the surface line defect to accommodate the tetrahedral anion. Differences between sulfate and molybdate substitutions can be mostly explained by the size of the anions. The molybdate oxyanion is more difficult to incorporate in the calcite bulk than the smaller sulfate oxyanion. However, when molybdate is substituted at the surface, the elastic cost is avoided because the oxyanion protrudes out of the surface and gains stability via the interaction with water at the interface, which in balance results in more favorable surface substitution for molybdate than for sulfate. The detailed molecular-level insights provided by our calculations will be useful to understand the chemical basis of S- and Mo-based speleothem records.

## 1 Introduction

Paleoclimate reconstructions are essential for understanding modern-day climate change but are hindered by a lack of instrumental environmental records. Long and continuous climate reconstructions therefore rely upon geological archives (e.g., marine and lake sediments, trees, and speleothems) of prevailing environmental conditions in the past. Input parameters for climate models simulating past climate variability require information obtained from various geological phenomena, including volcanic eruptions, which can trigger global cooling by disrupting natural transfer of solar radiation when the volcanic material enters the atmosphere.^1,2^ Identifying the magnitude of volcanic activity that occurred before modern scientific and historical records involves examining geological archives for chemical indicators (proxies). Ice cores,^3−5^ tree rings,^6^ and marine sediments^7^ are examples of widely exploited geological archives, which have the potential to capture and preserve geochemical indicators of volcanic activity. These archives hold geochemical proxies in naturally undisturbed states for millennia, allowing for environmental indicators to be matched to specific periods of Earth’s history. In recent years, speleothems have received increased research interest for use as a new paleovolcanic archive.^8−11^ These calcium carbonate mineral structures are highly stable and have advantages over ice cores, due to their usual proximity to many volcanic sites. Speleothems form in discrete layers primarily from cave drip water. Chemical impurities associated with geological and atmospheric conditions following a volcanic eruption can be detected in radiometrically dated subsections of speleothem minerals.^12,13^ Prevalence of certain impurities can be indicative of an environmental anomaly, such as alteration of atmospheric chemistry from a volcanic eruption. For example, Badertscher and co-workers have successfully matched elevated levels of sulfur, molybdenum, and bromine to the well-known Minoan volcanic eruption that occurred on Santorini, around 1600 BCE.^8^ In that study, trace amounts of these elements were measured in speleothem samples by X-ray fluorescence spectroscopy. A quantitative link between measured concentrations of impurity and the characteristics of the volcanic eruption (e.g. eruption magnitude or proximity to the speleothem), has not been established so far. The exact chemical nature of the trace element-bearing species is still not well understood either, although the chemical form and mechanism of incorporation are likely to be important considerations to improve the interpretation of trace element records obtained from speleothems.

The calcium carbonate in speleothems is mainly in the calcite form, with aragonite also present in some cases.^14−16^ Other calcium carbonate phases can also be found in speleothems but are very rare. Recently, we used computer simulations to study the lattice incorporation of both sulfur and molybdenum in the form of tetrahedral molybdate anions, in all the naturally occurring bulk phases of calcium carbonate.^17^ We found that both sulfate and molybdate anions are unstable in the bulk of calcite, aragonite, or vaterite, with respect to the formation of naturally occurring competing phases. In the case of sulfate ions, only the least-dense vaterite polymorph provides a somewhat stable host for lattice substitution at low concentrations. A preliminary calculation of substitution at the calcite (10.4) surface (ignoring dynamic aspects and assuming a simple interface with vacuum) showed that substitution at the surface, where there is more space to accommodate the tetrahedron anion, is energetically more favorable than in the bulk.^17^ If indeed trace elements are mainly incorporated at the surface or grain boundaries, then, the crystallinity of the calcium carbonate in the stalagmite will directly impact detected trace element concentrations. This effect is important to interpret the speleothem record and requires a more careful and detailed investigation, considering some of the complexity of mineral surfaces and interfaces, which is the purpose of the present work.

The (10.4) surface of calcite investigated here is very stable and typically the most prominent in natural calcite particle morphology,^18−20^ making it a good representative surface for the present study. Calcite growth occurs through the formation and motion of monolayer steps at the surfaces.^21,22^ This growth is well known to be inhibited by the incorporation of cation impurities (most notably Mg^2+^) at the steps.^23−26^ Meyer and subsequent citing articles have indicated that the sulfate anion is also a calcite growth inhibitor.^25^ It is therefore clear that these steps can provide a more favorable environment for the incorporation of impurities than the bulk or the perfect surfaces.^27,28^

We report here ab initio molecular dynamics (AIMD) simulations of the incorporation of sulfate and molybdate oxyanions both at terrace and step sites of the calcite (10.4) surface in contact with water, which is assumed to be non-dissociated. There is some experimental evidence for the presence of OH^–^ groups from water dissociation at calcite surfaces,^29−31^ and recent theoretical work based on cluster models has considered the interaction of H^+^ cations with calcite models under low-pH conditions.^32^ However, the nature and location of the dissociated species are still debated, and density functional theory (DFT) simulations indicate that water adsorbs without dissociation at the calcite (10.4) terrace and steps, requiring charged defects such as anion vacancies to dissociate.^33^ Therefore, in this work, we will only consider the interaction of the (10.4) surface with water in a non-dissociative scenario.

## 2 Methodology

The bulk phase of calcite, a trigonal crystal with the space group R-3C (167),^34^ was modeled using a 3 × 3 × 1 supercell of the hexagonal unit cell, to minimize interactions between trace elements and their periodic images (minimum distance between images around 15 Å). Slab models were used to represent the calcite/water interface. The slab for the (10.4) perfect surface consisted of four CaCO_3_ layers in the crystallographic direction perpendicular to the surface, which is the typical thickness used in the DFT simulation of this surface.^35,36^ Using static optimization test calculations, we observe that increasing the thickness to six molecular layers changed the substitution energy for sulfate by only ∼0.1 eV. Parallel to the surface, the model is infinite (periodic in 2D), and a 2 × 3 supercell of the rectangular surface unit cell was used, which led to a model with 48 CaCO_3_ formula units for the pure calcite slab and a minimum lateral distance between impurity images of around 15 Å in the substituted slabs. The gap between slabs was filled with water molecules at a density of 1 g cm^–3^.

In order to illustrate the role of surface defects in the incorporation of impurities, we also considered “imperfect” or stepped surfaces. To represent formation of pits on the calcite (10.4) growth termination, we created a vicinal (31.8) surface model, which corresponds to an acute step in the standard (10.4) terrace. The model has similar thickness and lateral dimensions as those of the terrace, with 52 CaCO_3_ formula units in the case of the pure calcite slab. In addition to the acute step, it would be possible, in principle, to consider other point defects (cation or anion vacancies) or line defects, such as obtuse steps, to understand their role in oxyanion impurity incorporation. However, the high computational cost of AIMD limits the number of defect models that can be practically simulated. Obtuse steps, which are not considered here, might in fact be able to accommodate some impurities more easily than acute steps^37^ but also exhibit more complex behavior due to faster kinetics of dissolution.^38^ We therefore limit our examination of the role of defects to the case of acute steps. A future detailed investigation of the effect of specific surface defects on the incorporation of these impurities will require the implementation of more efficient simulation approaches, either via the extension of existing force fields^39−41^ to include molybdate and sulfate species or perhaps by using modern on-the-fly machine learning force field generation from AIMD.^42^

Three compositions were investigated in each slab model: pure calcium carbonate, calcium carbonate with (SO_4_)^2–^/(CO_3_)^2–^ substitution, and calcium carbonate with (MoO_4_)^2–^/(CO_3_)^2–^ substitution. In each substituted case, a single carbonate ion was replaced with the relevant trace element-containing oxyanion. At the perfect surface, the substitution was performed at the top layer, at the solid/water interface. At the stepped surface, we considered substitution only at the apex of the step, as this has the most space to accommodate the ion impurity, maximizing the thermodynamic benefits of this surface topography. The vicinal surface cleavage left two symmetrically inequivalent sites at the apex of the step, but our tests revealed negligible energetic differences between these two anion sites; therefore, we proceeded to consider just one of them. As a reference phase, each anion (carbonate, sulfate, and molybdate) was placed into a ca. 15 Å box containing water molecules at a density of 1 g cm^–3^. Each anion in a box of water introduces a double-negative charge to the simulation cell; this was corrected using a homogeneous charge background.

The AIMD simulations were performed at constant volume and temperature using the canonical NVT ensemble implemented in the CP2K software package.^43^ To find the equilibrium volume and cell parameters at 300 K, while avoiding a computationally expensive constant-pressure (NPT) simulation at *ab initio* level, we followed a two-step procedure. First, we optimized the bulk structure statically (i.e., at 0 K and ignoring zero-point effects) using CP2K, at the same level of theory (see below) used for the finite-temperature simulations. To calculate the temperature correction to the 0 K cell parameters, we then performed NPT classical molecular dynamics simulations at 300 K, using a calcite 3 × 3 × 1 bulk supercell. The classical simulations were carried out using the GULP code,^44^ with the carbonates force field library by Fisler et al.^45^ Cell vectors simulated in GULP at 0 and 300 K were used to calculate the average linear thermal expansion from 0 to 300 K, which was then applied as a correction to the cell vectors optimized at 0 K using the CP2K code. Table 1 shows that the predicted cell parameters are in reasonably good agreement with crystallographic measurements. The negative thermal expansion in the *a* axis is consistent with previous reports.^46^

**Table 1:** Calcite Cell Vector Lengths Obtained from CP2K at 0 K and after Finite Temperature Correction in Comparison with Experimental Values

	T = 0 K	T = 300 K	exp. (297 K)^34^
a (Å)	5.22	5.07	4.99
c (Å)	17.59	17.74	17.06

In the AIMD simulations, electronic minimization was carried out using the Quickstep implementation of DFT,^47^ where the orbital transformation method was employed.^48^ All DFT calculations used the generalized gradient approximation in the form of the revised Perdew–Burke–Ernzerhof (revPBE) exchange–correlation functional.^49^ The revPBE functional has been shown to be particularly effective for describing the liquid structure of water, especially compared to the standard PBE functional.^50^ For representation of dispersion corrections, which is particularly important when simulating liquids, Grimme’s D3 corrections were applied.^51^ In all calculations, the short-ranged, double-zeta, molecule-optimized basis sets were used with polarization on heavy atoms (DZVP-MOLOPT-SR-GTH).^52^ The Goedecker–Teter–Hutter (GTH) pseudopotentials were used to represent atomic cores.^53^ The Nose–Hoover thermostat was used for the NVT simulations, with a time constant of 13 fs.^54^ A time step of 1 fs was used in all simulations, with the initial temperature set to 300 K. Each interface model was run for ca. 120,000 steps, giving a total simulation time of 120 ps. For the AIMD simulations of *E*[Ca_*n*_(CO_3_)_(*n*−1)_*X*O_4_]_(slab,aq)_, each time step required, on average, around 6–8 s on 576 cores of the UK national supercomputing service ARCHER. The AIMD simulation of interface models reported herein required ∼134,000 CPU hours (wall-clock time × number of processors).

We characterize the thermodynamics of surface incorporation using three interdependent parameters (any two of them determine the value of the third). The first one is the *surface/liquid exchange energy*, which is the energy required to exchange one carbonate and one impurity anion between the surface and the aqueous phase
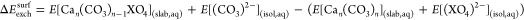
1where *E*[Ca_*n*_(CO_3_)_*n*−1_*X*O_4_]_(slab,aq)_ is the energy of the calcite/water slab model containing one (*X*O_4_^2–^) anion in a carbonate position, *E*[Ca_*n*_(CO_3_)_*n*_]_(slab,aq)_ is the energy of the pure calcite/water slab, and *E*[(CO_3_)2−]_(isol,aq)_ and *E*[(*X*O_4_)^2–^]_(isol,aq)_) are the energies of the isolated anions in water. In our calculations, there are *n* = 48 formula units in the terrace surface slab supercell and *n* = 52 formula units in the stepped surface supercell.

The second calculated parameter is the *bulk/liquid exchange energy*—the energy required to exchange one carbonate/trace ion from the calcite bulk to the aqueous phase
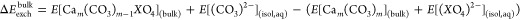
2where *E*[Ca_*m*_(CO_3_)_*m*-1_*X*O_4_] is the energy of the calcite bulk 3 × 3 × 1 supercell model containing one (*X*O_4_^2–^) anion in a carbonate position and *E*[Ca_*m*_(CO_3_)_*m*_] is the energy of the pure calcite unsubstituted supercell. Finally, the *segregation energy*, which is the energy required to exchange one carbonate/trace ion between the bulk and the (hydrated) surface of calcite, is given by
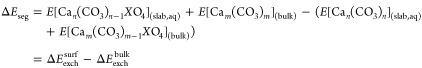
3

The absolute energies used in eqs 1–3 to define Δ*E*_exch_^surf^, Δ*E*_exch_^bulk^, and Δ*E*_seg_ were obtained from the average energies of the AIMD simulations after equilibration. In most cases, the first 20-30 ps of the AIMD simulation were required for equilibration, meaning that the production period consisted of 90–100 ps.

We also extracted radial distribution functions (RDFs) from the AIMD simulations. The RDF *g*(*r*) is defined in such a way that the number of atoms of the given type at a distance between *r* and *r* + d*r* from the central atom is proportional to g(*r*)d*r*, and the function is normalized so that g(*r*) = 1 when *r* → ∞. The RDFs were calculated using every frame from the final 20k AIMD steps and using bin sizes of 0.1 Å, up to a cutoff of 6 Å.

## 3 Results and Discussion

Table 2 summarizes the exchange and segregation energies obtained for molybdate and sulfate substitutions in calcite. The bulk/liquid exchange energies in calcite (1.08 and 1.62 eV for sulfate and molybdate, respectively) show the same trend as the bulk/vacuum exchange energies reported in our previous work (1.15 and 1.45 eV, respectively),^17^ that is, molybdate substitution incurred a greater energy penalty than sulfate substitution, due to the larger size of the former anion. Although the trend in the substitution energies was the same, the difference between sulfate and molybdate substitutions was found here to be different (larger) from what was reported in ref (17). This is partly because we now use a different thermodynamic reference phase, that is, oxyanions in water instead of oxyanions in vacuum, and partly because of the dynamic nature of the simulations here, in contrast with the static calculations in ref (17). We will see below that indeed, the incorporation of oxyanion impurities in calcite contains distinctive dynamic features that cannot be captured by static calculations, even for bulk substitution.

**Table 2:** Exchange and Segregation Energies of Molybdate and Sulfate Species in Calcite

	Δ*E*_exch_^bulk^ (eV)	Δ*E*_exch_^surf^ (eV)		Δ*E*_seg_ (eV)	
species	bulk	(10.4) terrace	(10.4) step	(10.4) terrace	(10.4) step
Sulfate	1.08	0.79	0.27	–0.29	–0.81
Molybdate	1.62	0.48	–0.07	–1.14	–1.69

Exchange energies at the (10.4) terrace are reduced when compared to equivalent bulk exchange energies, indicating the overall greater thermodynamic stability of the impurities at the surface. Unlike the bulk exchange process, molybdate incorporation at the terrace is more favorable than that of sulfate, which is surprising when considering that the former is a much larger species. These observations will be rationalized below, based on the discussion of the geometries of the substituted structures. Exchange energies at the step are even more favorable than at the terrace, indicating further thermodynamic favorability than bulk and terrace substitution. Like at the terrace, molybdate substitution is favored over sulfate substitution at the step, with a comparable energy difference of ca. 0.3 eV.

Overall, the stability of ion exchange in calcite with sulfate and molybdate follows the order: stepped surface > terrace > bulk. The tetrahedral oxyanion impurity does not fit in the planar carbonate anion site in calcite. As a result, significant lattice strain is caused in the bulk, which is reduced at surface regions due to the increased interfacial space around the substitution site. The step affords an even greater interfacial space than the terrace, which is why the trend in exchange energies at the bulk, terrace, and step follows the expected order. The crystallinity of the speleothem and factors affecting the stability of imperfect calcite surface topographies may therefore have a significant impact on detected concentrations of these trace elements in speleothems. Therefore, this should be an important consideration for the interpretation of impurity concentration data collected from speleothems.

**Figure 1:**
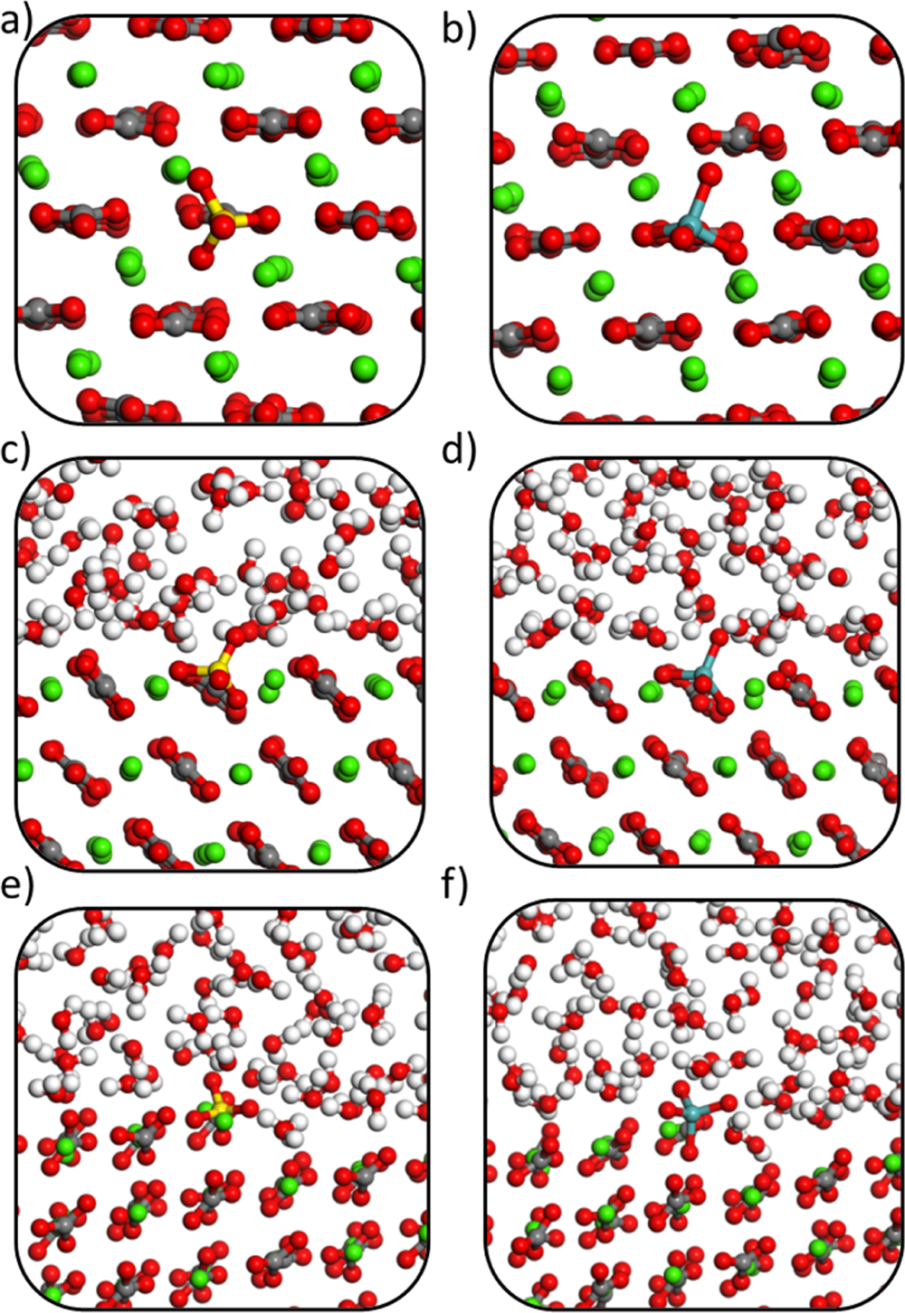
AIMD trajectory snapshots for calcite substituted with (a) sulfate at the bulk, (b) molybdate at the bulk, (c) sulfate at the terrace, (d) molybdate at the terrace, (e) sulfate at the step, and (f) molybdate at the step.

**Figure 2:**
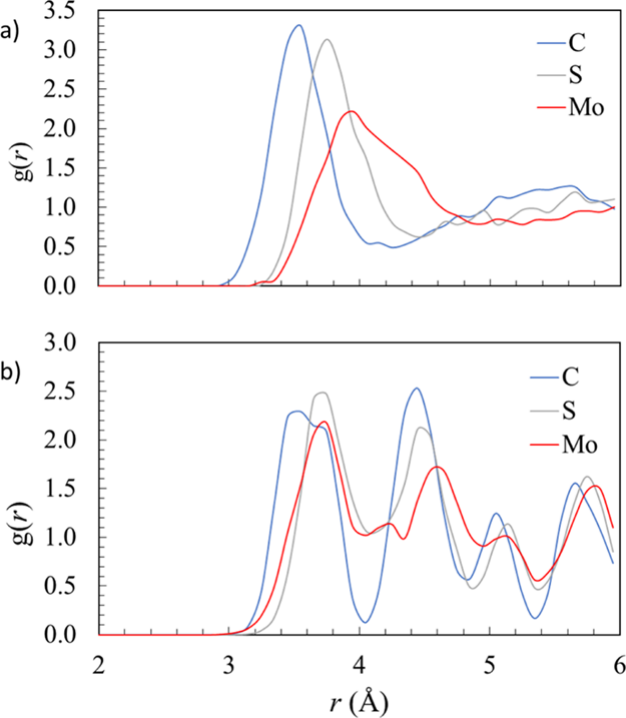
(a) RDF *X*–O_w_, that is, from the *X* = (C, S, or Mo) atom in the (*X*O_*n*_)^2–^ oxyanion to the oxygen atoms (O_w_) in water molecules, for the simulation of isolated oxyanions in water and (b) RDF *X*–O_c_, that is, from the *X* = (C, S, or Mo) atom in the (*X*O_*n*_)^2–^ oxyanion to the oxygen atom (O_c_) of the carbonate anions, for the simulation of bulk calcite.

To get further insights and rationalize the results from the thermodynamic analysis, we now discuss the AIMD trajectories and average geometries in equilibrium. Figure 1 shows snapshots from the AIMD simulations, taken after equilibration and at energies close to the equilibrium average. Figure 2a,b illustrates why the bulk of calcite is not well suited to accommodate the large tetrahedral oxyanion species, as the apical oxygen atom is unable to fit within the planar carbonate anion site. Interestingly, the tetrahedral oxyanions are found to rotate significantly with respect to the plane of the carbonate anions. For example, in the snapshot of the simulation of sulfate in bulk calcite (Figure 2a), two of the four oxygen atoms occupy the same oxygen positions as in the vacant carbonate ion, whereas the other two oxygen atoms are above and below the substituting plane. This contrasts with the equilibrium geometry found in our static DFT work,^17^ where three of the sulfate oxygen atoms occupied the three vacant carbonate oxygen sites with the fourth (apical) sulfate oxygen pointing out of the plane above the incorporation site. The observation of oxyanion rotation suggests that static calculations are not fully able to capture the behavior of tetrahedral oxyanion dopants in calcite, even for bulk simulations. This is important because static DFT (or force field-based) calculations are still the most widely used method for the simulation of impurities in carbonate minerals.

In contrast with the bulk substitution, when the oxyanion impurities are substituted at the surface, the apical oxygen atom can stick out, which not only decreases the elastic cost of the substitution but also leads to extra stabilization by a favorable interaction with water at the interface. To describe this effect in a more quantitative way, we turn to the calculated RDFs from the AIMD simulations.

We start with the analysis of RDFs for *X*–O_w_ (i.e., from the C, S, or Mo atom in the oxyanion to the oxygen atoms O_w_ in water molecules), for the simulation of isolated oxyanions in water, as reported in Figure 2a. The three anions have the same charge but different ion sizes (carbonate < sulfate < molybdate), as illustrated by the peak positions between 3 and 4 Å in the *X*–O_w_ RDFs. This means that the strength of the interaction with water is expected to follow the opposite trend (carbonate > sulfate > molybdate), in agreement with experimental hydration enthalpies; for example, the hydration of a carbonate anion is ∼2.6 eV more exothermic than the hydration of the larger sulfate ion.^55^ Compared with the case of sulfate, the first hydration sphere around molybdate is shifted right and broadened. There is also no clearly defined second hydration sphere, proving that polarization on the surrounding water is much weaker for molybdate. The weaker interaction of molybdate with water can be expected to lead to the faster kinetics of water exchange in and out the hydration spheres of the anions. This is consistent with our calculation of the mean residence time (MRT) of water molecules surrounding the sulfate and molybdate ions in aqueous solution. These are obtained as MRT = *t*_sim_ × CN_ave_/*N*_ex_, where *t*_sim_ is the AIMD simulation time, CN_ave_ is the mean coordination number for sulfate and molybdate, and *N*_ex_ is the number of exchange events involving the first hydration sphere during the simulation time (only those with a duration longer than 0.5 ps are counted). MRTs for sulfate and molybdate were calculated as 0.8 and 0.5 ps, respectively, which indicate that the hydration environments of both oxyanions are highly labile (comparable MRTs to those of the Cl^–^ ion)^56^ but more so for molybdate than for sulfate.

The *X*–O_c_ RDFs, that is, from the *X* = (C, S, or Mo) atom in the (*X*O_*n*_)^2–^ oxyanion to the oxygen atom (O_c_) of the carbonate anions, for the simulation of bulk calcite is shown in Figure 2b. The first peak at around 3.5 Å corresponds to *X*–O distances in the monolayer above and below the plane of the anion, and this clearly illustrates the structural impact of the impurity on the surrounding ions. Peaks for sulfate and molybdate are shifted right compared with those of carbonate, which is consistent with crystallographic strain and repulsion with neighboring carbonate ions, caused by the tetrahedral impurities. The second peak at around 4.5 Å corresponds to the *X*–O distances in the neighboring carbonate ions in the plane of the subject anion. Here, the sulfur and molybdenum peaks are shifted right, again indicating crystallographic strain and repulsion of the neighboring carbonate ions by the presence of the impurity. Where *X* = Mo, oxygen atoms are repelled most significantly, due to the large size of the molybdate anion.

At the surface, the unfavorable steric strain in the lattice caused by tetrahedral oxyanion substitution is reduced, as the apical oxygen atom can protrude from the solid surface rather than causing strain on neighboring ions in the crystal. This effect is further extended at the step where the substituting anion is afforded maximal interfacial space, meaning very low structural strain on the carbonate solid. At the step, three out of four oxygen atoms can protrude from the carbonate solid, while still allowing the anion to be chemically bound. This explains why overall, the surface substitutions are more favorable than the equivalent bulk substitutions.

**Figure 3:**
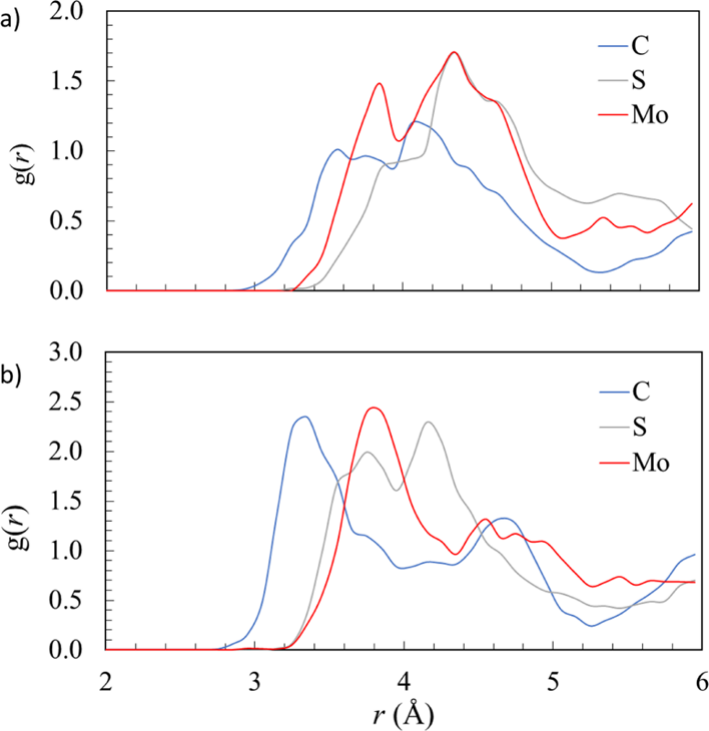
RDF *X*–O_w_, that is, from the *X* = (C, S, or Mo) atom in the (*X*O_*n*_)^2–^ oxyanion to the oxygen atoms (O_w_) in water molecules, for the simulations of oxyanion substitutions at (a) calcite terrace and (b) calcite step.

Comparison of RDFs for molybdate and sulfate at the terrace and step may be made from Figure 3a,b, which explains the lower exchange energies for both ions at the step than at the terrace, as reported in Table 2. When substituting the impurity at the terrace, there is an arc of around 180° which is in contact with the water (the other 180° is in contact with the lattice). When substituting at the step, however, the angle of the arc in contact with the water is around 270°, which allows for the formation of larger partial hydration spheres, which drive thermodynamic favorability. We discuss molybdenum as an example (sulfate may be assumed to behave in the same way but with effects less pronounced because of its smaller size). RDFs reported in Figure 3 show the partial hydration environment when molybdate is incorporated at the terrace and the step. From Figure 3, the first hydration sphere around molybdate at 3.8 Å is clearly better defined at the step (4b) than at the terrace (4a). This is because the molybdate ion at the step has more contact with liquid water, causing greater polarization of the aqueous layer and more favorable partial hydration spheres. A combination of decreased lattice strain and increased hydration sphere formation at the step when compared to the terrace explains why there is a thermodynamic preference for substitution at the step.

Another significant trend in the exchange energies (Table 2) is that molybdate appears more favorable than sulfate when substituting both surface sites, which is a reversal of the bulk incorporation energy trends. This means that despite greater steric strain caused by molybdate over sulfate, other factors are significant in driving the thermodynamic exchange energy. A possible explanation comes from the different levels of hydration of the substituting ions at the surface. As the molybdate ion protrudes more readily from the surface, aside from reducing lattice strain, this also allows for the formation of stronger partial hydration spheres. Hydration sphere formation is favorable and drives the thermodynamic process of substitution. Figure 3a reports RDFs showing the partial hydration spheres formed around sulfate and molybdate ions substituted at the calcite terrace. Molybdate shows a clear first peak at around 3.8 Å, while sulfate shows only a very small shoulder at this region. The explanation for this is that the molybdate ion protrudes from the surface more significantly than the sulfate, meaning that the central atom (Mo) is closer to water than the central atom (S) in sulfate, which is more deeply embedded into the lattice. Similar trends are reported in Figure 3b—RDFs showing partial hydration environments for sulfate and molybdate substituted at the calcite step. The molybdate shows a stronger, more well-defined first hydration sphere when compared to sulfate because it is repelled from the carbonate crystal more strongly and is therefore in closer contact with the surrounding water. This analysis provides more evidence to the notion that molybdate is more strongly ejected from the terrace while remaining chemically bound. Our proposed mechanism of stronger partial surface rehydration for molybdate over sulfate also gives another thermodynamic contributor to the relative favorability of molybdate substitution over that of sulfate at this site.

Finally, while the discussion given above focuses on the thermodynamics of impurity incorporation, the presence of sulfate and molybdate impurities is likely to significantly impact the kinetics of surface processes, including the dynamics of water adsorption/desorption from the surface and, at longer timescales, the dynamics of mineral growth/dissolution. These processes are beyond the scope of this work, and their consideration would require free energy calculations using, for example, metadynamics techniques, which are very computationally expensive for the size and number of models considered here. However, to gain some preliminary insights into the kinetics of water adsorption at the interface and the effect of molybdate and sulfate impurities on that kinetics, we have performed an analysis of the water exchange rate at the calcite–water interface, in the absence and presence of sulfate/molybdate impurities. We counted the number of water molecules going in and out of the first hydration spheres of surface Ca^2+^ cations, with a radius of ∼3 Å according to the Ca–water RDF. As in the analysis for the individual anions in water presented above, exchange events had to last for at least 0.5 ps to be counted, that is, rapid oscillations at the boundary were not included. The results are shown in Table 3. The rate of water exchange events increases from the pure carbonate surface to the sulfate-substituted one and then increases even more for the molybdate-substituted one. This behavior can again be rationalized in terms of the weaker polarization by the molybdate anion of the surrounding water molecules, compared with the case of sulfate, as discussed above for the cations of the isolated ions in water.

**Table 3:** Rates of Water Exchange Events at the Pure and Substituted (10.4) Terraces of Calcite (per Picosecond and Normalized by the Number of Surface Cations)

surface	water exchange events per ps
pure carbonate	3.19
sulfate-substituted	4.27
molybdate-substituted	4.56

## 4 Conclusions

We have presented an AIMD investigation of the incorporation of sulfate and molybdate as substitutional impurities in the bulk and surface of calcite. Results from this investigation indicate a strong tendency for ion exchange at the calcite/water interface regions of the speleothem when compared to exchange with the crystalline bulk. When comparing the incorporation of a given impurity species across the bulk, terrace, and step substitution sites, the ability of the solid to accommodate the tetrahedral oxyanion is still the key thermodynamic factor. In order to understand the differences between sulfate and molybdate substitution thermodynamics, we need to pay attention to other factors, for example, the partial hydration of the impurity at the calcite surface.

Bulk ion exchange of molybdate with liquid water is more thermodynamically unfavorable than that of sulfate, which is due to the increased lattice strain caused by the much larger molybdate ion. Exchange at both the terraced and stepped interfaces is more favorable for molybdate than for sulfate, which is a reversal in the trend observed for the two ions in the bulk. We rationalize this trend reversal by considering that the molybdate ion is protuding out of the solid surface more significantly due to higher lattice strain but is able to remain chemically bound to the solid due to its longer *X*–O bonds than sulfate. This proposed mechanism is supported by the analysis of RDFs indicating stronger partial rehydration of the molybdate impurity at the surfaces because the ionic center (Mo) is less deeply embedded in the carbonate solid, meaning closer proximity to the surrounding water than the analogous S atom. Although our analysis here is based on thermodynamics, the preferential substitution of the anion impurities at the surface and steps of calcite must also have important consequences for the growth kinetics. Calcite crystal growth is dominated by step dynamics, and the presence of impurities, particularly during extreme events such as volcanic eruptions, at the steps is known to stunt calcite growth.^24,37^ The interplay beyond thermodynamic and kinetic factors in this phenomenon is complex and beyond the scope of the present study.

Our calculations demonstrate and quantify the importance of surfaces and surface defects in the incorporation of oxyanion impurities in calcite. Since Mo and S impurities in speleothem calcites constitute a reliable record of past volcanic activity, the understanding gained of the factors controlling the concentrations of these impurities in calcite will be useful for a quantitative interpretation of speleothem records in the future.

## Notes

Input and output files from our simulations are available at the online repository https://doi.org/10.5281/zenodo.5041442.
